# Calciphylaxis of the penis and distal digits: a case report

**DOI:** 10.1186/s13256-021-03231-4

**Published:** 2022-01-18

**Authors:** Gordon Smilnak, Michael Jiang, Bijal Jain

**Affiliations:** 1 Northwestern University Feinberg School of Medicine, Chicago, IL USA; 2 Department of Medicine, Northwestern University Feinberg School of Medicine, Chicago, IL USA; 3 Division of Hospital Medicine at Jesse Brown VAMC, Northwestern University Feinberg School of Medicine, 820 S Damen Ave, Suite 7566, Chicago, IL 60612 USA

**Keywords:** Calciphylaxis, End-stage renal disease, Hemodialysis, Penis, Sodium thiosulfate

## Abstract

**Background:**

Calciphylaxis is a rare, often fatal disease resulting from calcification of dermal arterioles and capillaries. Usually diagnosed in patients with end-stage renal disease, this disorder typically presents as necrotic, nonhealing ulcers in acral or adipose areas. Here we report the case of an elderly man who was found to have calciphylaxis of the distal digits and penis, the latter of which is an uncommon site of disease that carries a particularly poor prognosis.

**Case presentation:**

A 73-year-old African American man with multiple medical comorbidities including dialysis-dependent end-stage renal disease presented with worsening painful, necrotic lesions on his glans penis and several distal digits over the last 2 months. The wound on the glans was foul smelling with overlying purulence and had been unsuccessfully treated with amoxicillin–clavulanic acid. Discovery of diffuse intravascular calcification on computed tomography, in addition to a markedly elevated calcium–phosphate product immediately prior to the onset of his ulcers, led to the diagnosis of calciphylaxis. The patient was initiated on sodium thiosulfate without improvement in his lesions, and he died 3 months later after another prolonged hospitalization.

**Conclusions:**

While calciphylaxis is a rare disease, involvement of the distal digits and especially the penis is even more uncommon and portends a particularly poor prognosis: 6-month mortality rates are reportedly as high as 70%. This suggests that prompt recognition and management of the disease is required; however, despite receiving standard therapy, our patient failed to experience improvement in his disease and instead developed several more fingertip ulcers at blood glucose sample points during his hospitalization. A corollary of the case presented here is the need for more effective management of calciphylaxis, especially for patients in whom uncommon sites, such as the penis, are involved.

## Background

Calciphylaxis is a rare disorder of intravascular calcification that is primarily observed in patients with longstanding end-stage renal disease (ESRD). Patients develop necrotic skin ulcers covered by black “eschars” that are typically found in areas of adiposity such as the abdomen and thighs; however, distal (digits, penis) and visceral (lungs, intestines) sites can also be involved. Patients often have an elevated serum calcium-phosphate product (> 70 mg/dL) and exhibit diffuse intravascular calcification on imaging [1]. Skin biopsy may provide definitive evidence of calciphylaxis; however, diagnosis is often made clinically in patients who present with the above findings [2].

There is currently no consensus on the optimal treatment for calciphylaxis; management is guided by observational studies that advocate for a multimodal approach involving wound care, proper analgesia, infection prevention, and correction of serum calcium and phosphate levels. A trial of sodium thiosulfate (STS) is also recommended for all patients with calciphylaxis owing to its reported success in several case series; however, there is a paucity of large-scale clinical trials to support this [3]. Even with appropriate management, calciphylaxis is a lethal disease: 6-month mortality rate is estimated to be 50%, with most patients dying from overwhelming infection [4]. Involvement of the penis, as in our patient, is a particularly poor prognostic indicator, with 6-month mortality rates as high as 70% [5, 6].

Here we present the case of an elderly male with ESRD and painful necrotic ulcers of his glans penis and digits. This case depicts a distribution of calciphylaxis that is uncommon and carries an exceptionally high mortality burden; it also highlights the need for further study of this condition.

## Case presentation

A 73-year-old African American man with a history of ESRD on hemodialysis, coronary artery disease status post-high-risk percutaneous coronary intervention requiring mechanical circulatory support 3 weeks prior, heart failure with preserved ejection fraction, hypertension, and diabetes, presented with necrotic lesions on multiple distal digits and the glans penis developing over the past 2 months. The patient denied any new exposures or trauma to the areas of ulceration and had no history of tobacco use. Of note, the lesion on his glans penis was recently treated with a 1-week course of oral amoxicillin–clavulanic acid without improvement.

**Fig. 1:**
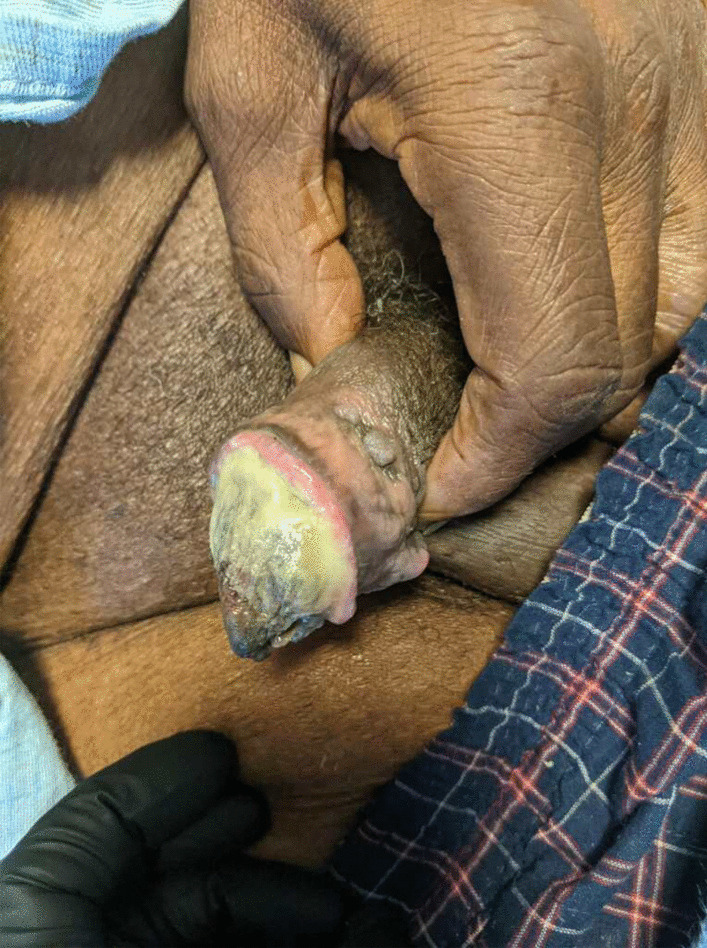
Image of the patient’s penis shortly after admission. Necrosis of the glans with overlying purulence can be seen

The patient was afebrile and had a blood pressure of 87/52 mmHg. Physical examination revealed several small, exquisitely tender, necrotic ulcers with overlying black eschars on multiple finger pads; a gangrenous distal left third toe; and a foul-smelling lesion on the glans penis with overlying purulence (Fig. 1). The digital lesions were cool to the touch and without surrounding edema or erythema. Examination was otherwise unremarkable except for a systolic ejection murmur that was noted previously.

Laboratory results obtained during admission were notable for a blood urea nitrogen of 40 mg/dL (reference range 7–21 mg/dL), potassium 5.1 mmol/L (3.5–4.7 mmol/L), calcium 9.1 mg/dL (8.5–10.1 mg/dL), phosphorous 1.5 mg/dL (2.5–4.9 mg/dL), parathyroid hormone (PTH) 104 pg/mL (18.4–80.1 pg/mL), albumin 2.9 g/dL (3.4–5.0 g/dL), white cell count 6.8 K/μL (4.0–11.0 K/μL), hemoglobin 7.3 g/dL (13.0–17.0 g/dL), and hemoglobin A1c 6.9% (≤ 5.6%). Upon chart review, his serum calcium and phosphorus were found to be 9.2 mg/dL and 9.0 mg/dL (product = 82.8 mg/dL) when the penile lesion first appeared.

**Fig. 2:**
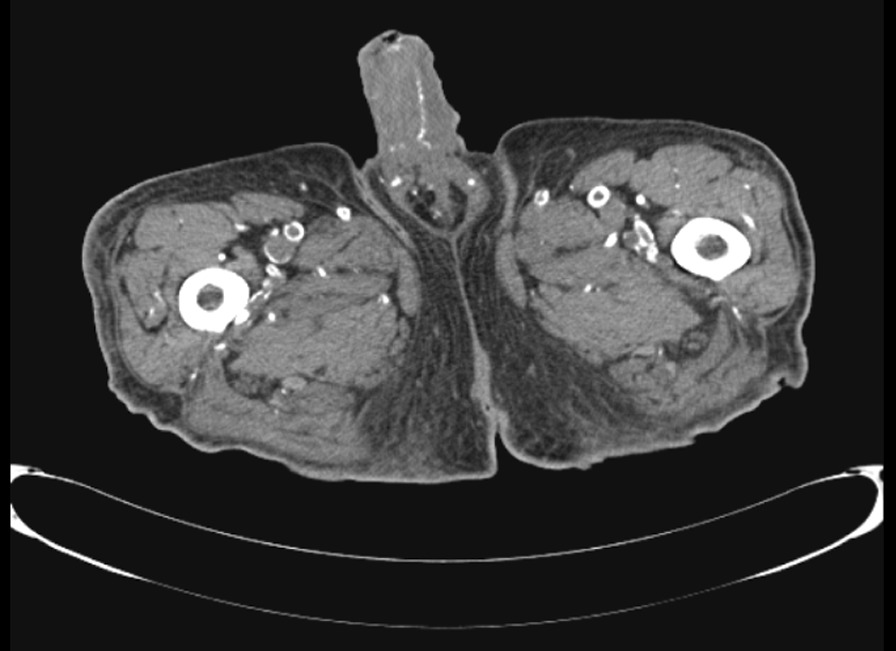
Computed tomography scan of the patient’s pelvis. Imaging revealed diffuse vascular calcification including the penile arteries, as shown. These radiographic lesions correlated with overlying skin necrosis

The patient underwent a computed tomography (CT) scan of the abdomen and pelvis, which revealed extensive intravascular calcification correlating with the ulcerating wound on the glans (Fig. 2). These radiographic findings, in a patient ESRD and recently elevated calcium and phosphate levels, yielded a clinical diagnosis of calciphylaxis. Treatment with thrice weekly sodium thiosulfate (25 g) during dialysis was initiated. A confirmatory skin biopsy performed later in his hospital course was inconclusive.

Although the patient tolerated treatment without appreciable side effects, he experienced no improvement in the digital or penile lesions; in fact, he developed several more painful, necrotic ulcers corresponding with finger-stick blood glucose sample points. He was discharged home with plans to continue regular STS during dialysis. On follow-up, his wounds remained painful but showed no further progression; unfortunately, he died 3 months later after another prolonged hospitalization.

## Discussion

Calciphylaxis is a devastating disease that results from significant calcification of the tunica media of dermal arterioles and capillaries. The exact mechanism of pathogenesis is unknown, but hyperparathyroidism, hypervitaminosis D, and elevated serum calcium and phosphorus have all been implicated [7]. It is thought that calcium deposition and subsequent endothelial injury promotes formation of microthrombi, which reduces luminal caliber leading to ischemia and necrosis of overlying tissues.

Calciphylaxis is usually diagnosed in patients with dialysis-dependent ESRD; other risk factors include diabetes mellitus, autoimmune disease, malignancy, kidney transplant, and hypoalbuminemia [7, 8]. The annual incidence in patients with ESRD is approximately 0.04–4% and thought to be increasing [1, 9]. Prevalence of calciphylaxis has been less well studied and is likely underestimated; one cross-sectional study estimated the prevalence in ESRD to be 4.1% [10]. The average age at diagnosis is between 50 and 70 years, with roughly two-thirds of patients being women [1, 11]. While calciphylaxis has been conventionally thought to develop over areas of adiposity, a 2019 meta-analysis of 856 cases found that the distal lower extremities were the most common site of involvement (55%), followed by the proximal lower extremities (40%) and then the trunk (31%) [2, 12]. Upper extremity involvement, as in our patient, was only seen in approximately 10% of all cases. Due to its rich vascular network, calciphylaxis of the penis is even less common: a 2018 review estimates that as few as 50 cases have been reported in the literature [13, 14].

Lesions in calciphylaxis may begin as indurated nodules or plaques resembling livedo reticularis, before progressing to necrotic ulcers with or without eschars [15]. However, initial presentation can vary significantly, so diagnosis requires a high degree of suspicion in the proper clinical context. Other features that may suggest presence of the disease include obesity, hyperparathyroidism, and an elevated (> 70 mg/dL) serum calcium–phosphate product in the months leading up to appearance of the lesions, as seen in our patient [16]. Further diagnostic testing is not required if the presentation is typical; however, if imaging is obtained, it may reveal intravascular or soft tissue calcification in corresponding areas [17]. Skin biopsy can provide definitive evidence of calciphylaxis, but is often reserved for ambiguous cases since it can predispose to wound complications or be nondiagnostic, as it was in this patient [18]. Importantly, some sources even argue that biopsy is contraindicated in penile calciphylaxis because of low diagnostic yield, as well as the particularly high risk of sepsis or acceleration of necrosis [19, 20].

A major challenge in management of calciphylaxis is the lack of consistent supporting evidence for a first-line treatment. Sodium thiosulfate (STS) is a drug that may possess beneficial vasodilatory and antioxidant effects and has been associated with longer survival in several case series; however, subjects in these studies were often managed with complex treatment regimens, making it difficult to assess the efficacy of STS monotherapy [2, 21]. Furthermore, a 2019 meta-analysis failed to demonstrate an improvement in wound progression or mortality from calciphylaxis through use of STS [12]. Nevertheless, a trial of this medication is recommended in all patients for at least 1 month [3].

In addition to STS, management of calciphylaxis is multifaceted and involves appropriate analgesia, wound care, infection prevention, and optimization of dialysis [22]. Abnormalities in serum calcium and phosphate should also be corrected through use of binders such as sevelamer, with a goal product of < 55 mg/dL [6]. Cinacalcet, a calcimimetic, is considered in cases of significant hyperparathyroidism (PTH > 300 pg/mL) and thus was not indicated in this patient [12]. If superimposed infection is suspected, as in our patient, empiric antibiotics with activity against methicillin-resistant *Staphylococcus aureus* and anaerobes should be initiated promptly given the high associated mortality risk. Despite theoretical benefits, anticoagulation does not play a major role in management; in fact, warfarin therapy has been established as a risk factor for calciphylaxis, possibly through inhibition of vitamin K-dependent regulatory proteins that prevent vascular calcification [7, 23].

Experimental therapies such as hyperbaric oxygen and pentoxifylline have been explored, with some success, in treating resistant calciphylaxis but continue to lack large-scale supporting evidence [7, 15]. Surgical management, such as revascularization procedures and partial or total penectomy, has also been described in select cases of penile calciphylaxis but does not appear to confer a survival benefit [13, 14].

Even with prompt initiation of treatment, calciphylaxis portends a dismal prognosis. The disease has a 6-month survival rate of approximately 50%, with disseminated infection continuing to account for most deaths [9, 24]. Of note, mortality is roughly tripled among patients receiving hemodialysis, such as the patient presented here [4]. Penile calciphylaxis appears to be especially resistant to accepted treatments and has a 6-month mortality rate of up to 70% [5, 6]. Reasons for this worsened prognosis are not fully understood, but it is thought that involvement of the rich network of penile arterioles and capillaries implies more systemic disease [13, 25]. This patient’s multisite presentation and failure to improve with standard management indicate that he likely had advanced calciphylaxis.

One additional unique aspect of this case was the subsequent development of painful, necrotic ulcers at fingertip blood glucose sample points. This presents yet another challenge in the management of calciphylaxis, since many patients have diabetes and poor glucose control may be a risk factor for worse disease [26, 27]. Though involvement of the distal upper extremity is not as prognostically worrisome as penile calciphylaxis, this case serves as a reminder of the importance of mitigating iatrogenic trauma in susceptible patients [11]. Glucose monitoring was eventually discontinued in this patient and successfully prevented progression of his fingertip wounds.

## Conclusions

Overall, this case highlights the particularly poor prognosis associated with penile calciphylaxis. It also underscores the need for more effective management of the disease, especially in patients with multiple comorbidities.

## Data Availability

Data sharing is not applicable to this article as no datasets were generated or analyzed during the current study.

## Abbreviations

ESRD: End-stage renal disease
STS: Sodium thiosulfate
PTH: Parathyroid hormone
CT: Computed tomography
